# Leptin’s Pro-Angiogenic Signature in Breast Cancer

**DOI:** 10.3390/cancers5031140

**Published:** 2013-09-06

**Authors:** Ruben Rene Gonzalez-Perez, Viola Lanier, Gale Newman

**Affiliations:** Department of Microbiology, Biochemistry and Immunology, Morehouse School of Medicine, 720 Westview Dr. SW., Atlanta, GA 30310, USA; E-Mails: vlanier@msm.edu (V.L.); gnewman@msm.edu (G.N.)

**Keywords:** leptin, breast cancer, tumor angiogenesis, Notch, IL-1, VEGF, VEGFR2, NILCO, Leptin Peptide Receptor Antagonist

## Abstract

Obesity is linked to increased incidence of breast cancer. The precise causes and mechanisms of these morbid relationships are unknown. Contradictory data on leptin angiogenic actions have been published. However, accumulating evidence would suggest that leptin’s pro-angiogenic effects in cancer play an essential role in the disease. Leptin, the main adipokine secreted by adipose tissue, is also abnormally expressed together with its receptor (OB-R) by breast cancer cells. Leptin induces proliferation and angiogenic differentiation of endothelial cells upregulates VEGF/VEGFR2 and transactivates VEGFR2 independent of VEGF. Leptin induces two angiogenic factors: IL-1 and Notch that can increase VEGF expression. Additionally, leptin induces the secretion and synthesis of proteases and adhesion molecules needed for the development of angiogenesis. Leptin’s paracrine actions can further affect stromal cells and tumor associated macrophages, which express OB-R and secrete VEGF and IL-1, respectively. A complex crosstalk between leptin, Notch and IL-1 (NILCO) that induces VEGF/VEGFR2 is found in breast cancer. Leptin actions in tumor angiogenesis could amplify, be redundant and/or compensatory to VEGF signaling. Current failure of breast cancer anti-angiogenic therapies emphasizes the necessity of targeting the contribution of other pro-angiogenic factors in breast cancer. Leptin’s impact on tumor angiogenesis could be a novel target for breast cancer, especially in obese patients. However, more research is needed to establish the importance of leptin in tumor angiogenesis. This review is focused on updated information on how leptin could contribute to tumor angiogenesis.

## 1. Introduction

The alarming increase in obesity and its yet unveiled mechanistic relationships with the development of several cancers are currently both scientific and medical challenges. Several research laboratories are looking for answers explaining these fatal relationships. Intense research is required to identify the molecular mediators and specific biological and genetic conditions causing obesity-related cancer [1,2,3]. Obesity is a state of mild chronic inflammation showing altered patterns of inflammatory cytokines [4,5]. Obese individuals also have higher levels of estrogen that increases the growth of endocrine responsive cancers in particular, breast cancer. Elevated long term estrogen exposure is a major risk factor for cancer in hormone dependent organs [6]. However, not all breast cancers respond to estrogens. Estrogen and progesterone unresponsive breast cancers are mostly dependent on growth factors (*i.e.*, insulin, insulin-like growth factor-I) and adipokines (*i.e.*, leptin) [7,8,9].

Leptin’s most known role is related to energy balance. Levels of leptin correlated to adipose tissue and body weight. In normal weight individuals, leptin controlled appetite and energy expenditure through complex signaling actions at hypothalamic levels. However, obese individuals have high levels of leptin but cannot control their appetite. Therefore, obesity is characterized as a “leptin resistant state” [10]. High plasma levels of leptin have been linked to breast cancer development [11].

Leptin receptor, OB-R, and the estrogen receptor, ER, are co-expressed in breast cancer, which suggests an interaction between leptin and estrogen systems to promote breast carcinogenesis [12]. Estrogen may influence leptin synthesis in a tissue and cell type specific fashion [13]. However, antiestrogens induced leptin synthesis and release in adipocytes [14]. Conversely, leptin transactivated ER [15] and increased the expression of aromatase in breast cancer cells [16].

An essential process for the growth of solid tumors is the constant supply of oxygen and nutrients needed for the rapidly proliferating cancer cells [17]. To achieve this, tumor cells secreted angiogenic factors and induced other cells to produce them as well. These factors are required by endothelial cells to proliferate, differentiate and form blood vessels, a process involved in the development of tumor angiogenesis [18].

Both physiological and pathological angiogenesis involves the effects of several pro- and anti-angiogenic (angiostatic) molecules, allowing endothelial and mural cells (pericytes and vascular smooth muscle cells) to form new vessels from existing ones. In addition, tumor vasculogenesis, formation of new vessels, also played a role in the formation of the tumor vasculature, which is mediated by angioblasts or stem cells [19]. Remarkably, adipose tissue, adipocytes and adipose stromal cells, also secrete several factors that promoted angiogenesis, HGF, TFG-β, VEGF, TNF-α, angiogenin, resistin, leptin, and angiostatic molecules, adiponectin [20,21,22,23,24,25]. Several studies reported that adipogenesis and angiogenesis are tightly correlated and showed adipose tissue mass decreased when an antiangiogenesis drug was given to diet induced obese mice [26].

An important factor released from adipose tissue impacting tumor angiogenesis is leptin, an adipokine, that promoted the proliferation and angiogenic differentiation of endothelial cells *in vitro* and *in vivo* [27,28]. Specific distribution of leptin and OB-R, suggested an important autocrine and paracrine role for leptin in human adipose tissue. OB-R in human white adipose tissue was not restricted to adipocytes but was present in resident endothelial and immune cells within adipose tissue [29].

As are other cytokines, leptin is a ubiquitous and pleiotropic molecule, which is involved in a growing number of processes, *i.e.*, energy balance, inflammation, reproduction, angiogenesis and cancer [11,30,31]. The ER upregulated a number of genes involved in angiogenesis and vasculogenesis [32,33] and leptin crosstalk to ER could contribute to the development of estrogen-responsive breast cancer and tumor angiogenesis. However, this is a subject that needs more research. Furthermore, leptin induced IL-1, Notch, VEGF/VEGFR2, matrix metalloproteinases (MMPs), MMP inhibitors (TIMPs) and integrin expression in diverse tissues [34,35,36], which could further contribute to leptin pro-angiogenic effects. Overall, leptin plays roles in physiologic [23,27,28] and abnormal angiogenesis atherosclerosis and cancer [11,31,36].

## 2. Angiogenesis and Tumor Angiogenesis

Angiogenesis is a natural process that occurs during embryogenesis, wound healing, and hormonal processes such as, menstruation, ovulation, and pregnancy. Angiogenesis involves the formation of new blood capillaries from preexisting microvessels and venules. This process comprises a cascade of events driven by growth factors.

VEGF-family is the main angiogenic factor for endothelial cells. The VEGF family of ligands is composed of VEGF-A, B, C, D and E. These ligands bind a family of receptors, VEGFRs, which is composed by three members, VEGFR1 (Flt-1), VEGFR2 (KDR/Flk-1) and VEGFR3 (Flt-4). These are transmembrane tyrosine kinase receptors with some degree of binding-affinity for VEGF ligands. VEGF-A, the principal ligand, can bind VEGFR1 and VEGFR2 to regulate the formation of both blood (angiogenesis/vasculogenesis) and lymphatic (lymphoangiogenesis) vessels [37].

VEGFR2 directly regulates tumor angiogenesis [37]. VEGFR2 is commonly recognized as mediating VEGF-induced responses. VEGFR2 also binds VEGF-C, VEGF-D and VEGF-E, whereas VEGFR1 can bind to VEGF-B and placental growth factor, (PIGF) [38,39,40,41]. VEGFR2 is considered as the earliest marker for endothelial cell development. VEGF/VEGFR2 activation induced a signaling cascade, which leads to the activation of endothelial cells and the degradation of the basement membrane. These cells proliferate, migrate and secrete factors, such as MMPs, for extra cellular matrix breakdown and the new blood vasculature is stabilized by perivascular cells [42]. In addition, non-vascular endothelium-specific factors contributed to blood vessel formation, *i.e.*, platelet-derived growth factor, PDGF and TGF-β families [43].

The release of pro-angiogenic factors such as VEGF, PDGF, PIGF [42,44], and leptin [27,28,45] can cause endothelial cell activation, proliferation, and migration. Recruitment of inflammatory cells also significantly contributed to adipose neovascularization. Activated macrophages produced potent angiogenic factors such as TNF-α, VEGF, FGF-2, IL-1β, IL-6, and IL-8. It has been found that increased leptin levels in the blood of obese patients can lead to excessive angiogenesis [46]. Pathological angiogenesis associated with wound healing often occurred subsequent to an inflammatory response that includes the secretion of cytokines such as TNF-α [47].

Abnormal or pathological angiogenesis occurs during cancer, retinopathies, diabetic neuropathies, and endometriosis [42]. Extensive laboratory data suggested that angiogenesis plays an essential role in breast cancer development, invasion, and metastasis [48]. It has been long recognized that the angiogenic process is an essential step in the progression of a variety of solid tumors [17]. Results of experimental animal tumor models and observations of human cancer involved the induction of angiogenesis and was a discrete component of tumor evolution activated during the early premalignant stages of tumor development [49].

Tumor angiogenesis is triggered by the metabolic needs of the tumor. The angiogenic switch is a discrete step that is required for tumor progression by which the tumor induces an independent vascular network. Tumor vessels are quite imperfect, which is reflected in their abnormal wall organization, branching shapes and scarce number of mural cells [19]. Tumor vessels are characterized by excessive leaking and increased permeability [19,50,51].

The main promoters of tumor angiogenesis are hypoxia and necrosis, which induce the release of pro-angiogenic factors to develop the new vascular network from existing venules and capillaries [49]. Strikingly, the angiogenic switch preceded the transformation of mammary hyperplasia to malignancy [52]. The creation of new blood vessels allows the tumor cells to escape from their hypoxic environment and enter the blood stream to metastasize in a new tissue environment.

## 3. Leptin and OB-R Expression and Signaling

Leptin and OB-R are mainly expressed in the hypothalamus and adipose tissue [30]. These molecules are weakly expressed in non-malignant cells, but overexpressed in cancer cells in the colon [53,54], liver [55], brain [56,57] and breast [11,58,59,60]. Leptin and OB-R are expressed in more than 80% of breast cancer tissue [61] according to immunohistochemistry results in which only cancer cells were evaluated for antigen expression, however, the expression of leptin or OB-R in stroma was not determined. In whole tissue breast cancer tissue arrays (n = 75; Pantomics, Inc., Richmond, CA, USA), leptin and OB-R stained cells were detected in 46%−70% of samples [62].

Leptin is a small adipokine (precursor: 167 amino acids and mature protein: 146 amino acids, 16 kDa) encoded by the obesity (ob or LEP) gene. Leptin shows hormonal features and highly conserved sequences in mammals. Only conservative amino acid substitutions within leptin’s potential binding sites to its receptor are found among all leptin molecules reported [63]. Leptin showed no homology with any other proteins, exists as a unique isoform, and binds with high affinity to leptin receptor OB-R. In contrast, several isoforms of the membrane-bound leptin receptor, OB-R, (coded by db gene) were found in diverse tissues. OB-RL or OB-Rb is the longest and fully functional isoform. Other isoforms (OB-Ra-f) showed truncated cytoplasmaic tails of different lengths and were derived from posttranscriptional splicing of OB-RL. OB-R belongs to the class I cytokine receptor super-family, which lacks autophosphorylation activity, but needs auxiliary kinases for activation. OB-R only bound leptin and leptin can only bind OB-R. Therefore, strict biunivocal binding-affinity and activation of leptin/OB-R complex have been suggested [11]. However, leptin binding to OB-R triggered diverse signaling pathways according the presence of two loci in the OB-R cytoplasmic tail. Leptin binding to OB-RL containing Box 1 and 2, induced canonic (JAK2/STATs, MAPK/ERK1/2 and PIK-3/AKT) and non-canonic (PKC, AMPK, p38, JNK) signaling pathways. Leptin binding to shorter isoforms of OB-R lacking Box 2 did not activate JAK/STATs signaling [64,65].

## 4. Regulation of Leptin Expression

Leptin plays a major role in the regulation of appetite and energy balance. Serum levels of leptin were increased due to the enlargement of adipocytes. During adipocyte differentiation leptin synthesis and secretion was regulated by several factors [66]. Insulin and glucocorticoids synergistically upregulated the transcriptional expression and secretion of leptin over the long term. Increased levels of leptin in obesity could be linked to chronic hyperinsulinemia and increased cortisol turnover. In turn, fasting lead to a gradual decline of serum leptin that was probably attributable to insulin decrease and the ability of catecholamines to reduce leptin expression [67].

Leptin is an inflammatory cytokine, therefore, it has a complex signaling and regulatory crosstalk with other inflammatory cytokines [31]. Earlier studies suggested that IL-1β is essential for leptin induction by both lipopolysaccharide and turpentine in mice, while IL-6 was not [68]. Moreover, IL-1 and TNF-α were inducers of leptin expression in mice and hamsters [69]. In breast cancer, IL-1 increased leptin expression in stromal cells recruited into the tumor microenvironment [70].

Additionally, estrogen affected leptin expression in several tissues [71]. The co-expression of OB-R and ER in breast cancer suggested a potential crosstalk between them that could induce breast carcinogenesis [60]. Antiestrogens stimulated the synthesis and release of leptin in the adipocytes [72].

There is limited information available on the transcriptional pathways regulating adipocyte-specific expression of leptin. Leptin gene regulation is complex and involves the effects of C/EBP, SP1, AP1, GR, and CREB and, as well adipocyte determination differentiation dependent factor 1/sterol regulatory element binding protein 1 (ADD/SREBP1c), which binds a classic TATA box and E-box element [73,74,75]. The decrease of ADD/SREBP1 under fasting conditions parallel the regulation of fatty acid synthase (FAS), leptin [75], C/EBP and AP1 transcription factors [73]. Recently, FOS2L was found to bind and regulate the adipocyte-specific cis-element upstream of the leptin gene in human fat cells. FOS2L was suggested as a critical regulator of leptin expression in adipocytes [76].

Hypoxia, a key signal for the induction of angiogenesis, increased the expression of hypoxia-inducible factor (HIF-1α), which is overexpressed in poorly differentiated breast cancer compared to well-differentiated carcinoma and nonmalignant breast tissue. The expression of HIF-1α was associated with increased expression of leptin and VEGF [77]. Indeed, leptin levels were increased under hypoxia in trophoblasts [78] and breast cancer [79] via HIF-1α. The HIF/p300 binding region of leptin gene contains four hypoxia-response elements and three GC-rich regions [79]. In breast cancer cells, HIF/p300 complex bound the upstream leptin regulatory sequences of the proximal leptin gene promoter. Hypoxia induced a significant reduction of perixome proliferator-activated receptor gamma (PPARγ) mRNA and upregulated leptin in pre-adipocytes [77]. This confirmed the earlier reported effects of antidiabetic thiazolidinediones. These drugs down-regulated leptin gene expression by mechanisms that may involve induction of PPARγ binding to the leptin receptor cis-regulatory elements (C/EBP and AP1 sities) [66]. In contrast, endogenous activators of PPARγ, such as fatty acids and prostaglandins, induced leptin. PPARγ could act as an adipocyte-specific factor facilitating transcription factor FOS-like antigen 2 (FOSL2; belonging to AP-1 transcription factor family) binding during differentiation [76].

Insulin also induces leptin expression in breast cancer cells by increasing HIF-1α and SP1 binding to the leptin promoter. These insulin effects were linked to the activation of ERK 1/2 and PI-3K signaling pathways [80]. Physiological concentrations of insulin increased transcriptional and translational expression and secretion of leptin in fibroblasts via a PPARγ independent mechanism [67]. In colon cancer cells (HT-29), EGF-induced PI-3K/STAT3 signaling was suggested as an essential pathway regulating VEGF and leptin expression. In these EGF-responsive colon cancer cells, EGF induced STAT3 binding to VEGF and leptin promoters and stimulated leptin and VEGF mRNA and protein synthesis [81].

Epigenetics changes could also play a role in leptin gene regulation. An inverse relationship between methylation and leptin expression has been reported. This relationship was accompanied by lower methylation density (8%) in visceral adipocyte fraction compared to the stromal vascular fraction of white adipose tissue and liver (18%, 21%; respectively). However, weight-loss induced changes in leptin expression did not seem to be methylation-dependent [82].

## 5. Leptin’s Impact on Tumor Angiogenesis

It was earlier suggested that leptin from adipocytes may act locally in a paracrine manner on endothelial cells expressing OB-R. Vascular endothelial cells expressed OB-R, and responded to leptin by promoting angiogenesis, proliferation and reducing apoptosis [27,28,45]. Leptin acted on endothelial cells causing fatty acid oxidation and angiogenic differentiation [27,28]. Leptin played a critical role in the maintenance and regulation of vascular fenestrations in the adipose tissue, increased vascular permeability and synergistically induced angiogenesis together with two notable angiogenic factors: VEGF and FGF-2 [83]. Leptin’s ability to promote angiogenesis is comparable to those obtained from VEGF actions [27,45]. Indeed, leptin mediated angiogenesis *in vivo* in human atherosclerotic aorta and also affected angiogenesis in peripheral tissues [36]. Long and short isoforms of the OB-R were coexpressed in various hemopoietic organs, and a subset of cells from these tissues bound leptin [84].

In addition to increasing the proliferation of endothelial cells [27,28,45,85], leptin also caused the proliferation of fibroblasts [86] and diverse malignant cells. In these cells, leptin induced pro-angiogenic, inflammatory and mitogenic actions that were reinforced through crosstalk with several cytokines/growth factors [11,31,87]. Remarkably, it was recently affirmed, the potential contribution of stroma fibroblasts to the paracrine effects of leptin on cancer cells. Cancer-transformed and “educated” fibroblasts played an essential role contributing to the progression of cancer via leptin signaling crosstalk [88].

Accumulated evidence suggested that leptin signaling gave an additional advantage to breast cancer progression by upregulating VEGF/VEGFR2 before hypoxia occurred [89]. VEGF/VEGFR2 directly regulated tumor angiogenesis and also worked as an essential autocrine/paracrine process for cancer cell proliferation and survival [37]. Additionally, conditioned medium from glioblastoma cell lines, containing leptin, significantly stimulated proliferation and increased human umbilical endothelial cells (HUVEC) tube formation. These effects were blocked by Aca 1, an OB-R peptide antagonist, and by SU1498, which inhibited the VEGFR2 [85].

Inflammatory cells (*i.e*., macrophages) contributed to breast cancer angiogenesis [46]. Primary effects of inflammatory cells in tumors were more likely to contribute to cancer cell growth, progression, and immunosuppression than to elicit an effective antitumor response [90]. Leptin attracted macrophages and other inflammatory cells expressing OB-R to the tumor microenvironment, which in turn promotes angiogenesis [11].

Data from paracrine effects of leptin *in vitro* may still be considered speculative since a study on tumor development using the fatless A-Zip/F1 mouse model showed a different conclusion. These mice lacked leptin and other adipokines, but tumor growth was linked to effects elicited by insulin resistance and inflammation. However, no change in tumor development was found when these fatless mice were crossed to the mouse mammary tumor virus-Her2/neu transgenic mouse model of mammary cancer (MMTV-Her2). This data delineated the complexity of tumor angiogenesis and the relative role of other factors in absence of adipokines [91].

It was earlier reported that adipose tissue growth is angiogenesis dependent and leptin did not play an essential role in angiogenesis. This data was obtained from studies conducted in the leptin deficient mutant (*ob/ob*) mouse model [92]. *Ob/ob* mice without leptin are indeed obese and show adipose tissue angiogenesis. A marked vascular remodeling was evident in adipose tissue sections from mice receiving anti-angiogenic therapies. Moreover, TNP-470 (an angiogenesis inhibitor) and leptin similarly reduced fat mass relative to controls (*p* ≤ 0.01) resulting in similar decreases in percent body fat. Food intake in mouse treated groups was comparable and significantly less than those found in control mice (*p* < 0.0005). However, it is important to note that leptin is a known regulator of adipose tissue size. Therefore, some of the leptin actions on adipose size could be related to decreased food intake. In addition, exogenous leptin induced the reduction of endothelial cell proliferation, which was accompanied by increased apoptosis [92].

It is known that apoptotic mechanisms could be involved in the actions of specific anti-angiogenic factors. Remarkably, leptin has been linked to adipose tissue apoptosis. Leptin mRNA was higher in Sc than in Om adipocytes [93,94]. This relationship was the inverse for cellular inhibitor of apoptosis, the protein-2 (cIAP2) mRNA. Depot-specific differences (in leptin and cIAP2) appeared to play a role in the regulation of apoptosis of adipose tissue [94]. In addition, reduction of adipose tissue through apoptosis has been observed after intra-cerebroventricular administration of leptin in rats. Adipose tissue of leptin-treated rats demonstrated characteristic features of apoptosis, including internucleosomal fragmentation of genomic DNA, elevated levels of DNA strand breaks and a reduction in total DNA content and cell volume. However, leptin’s effect on apoptosis process differed in other cell types. In myeloid leukemic cell lines, leptin had proliferative and anti-apoptotic properties. Ob-protein reduced apoptosis induced by cytokines in these cell lines (For Review see [30]). Whether the endothelium functions similarly in non-neoplastic and tumor tissue growth is not completely known. Leptin anti-angiogenic actions in *ob/ob* mice [92] need to be carefully considered and their significance could differ in neoplastic *versus* non-neoplastic tissues.

Leptin deficient and obese (*ob/ob*) mice implanted with syngeneic mouse melanoma cells (B16F10) did not show positive correlation between leptin levels and tumor growth. Moreover, in these mice tumor VEGF was independent of host plasma leptin levels. Intriguingly, reduced melanoma growth was found in lean, pair fed leptin deficient *ob*^−/−^ mice, compared to tumors from lean wild type or *ob*^+/−^ mice, which have similar body weights but higher leptin levels. These observations suggested that leptin deficiency greatly attenuated melanoma tumor growth while high leptin levels accelerated tumor growth. Additionally, obese MC4R^−/−^ (melanocortin receptor 4 knockout) mice with high plasma leptin levels showed higher development of melanoma compared to lean controls. The authors suggested that leptin is not essential for melanoma growth but may accelerate tumor growth [95]. In contrast, a more recent study used the same mouse melanoma cell line implanted into C57BL/6J mice, showed a significant increase of tumor growth (188%) after exogenous leptin supply. Furthermore, it was suggested that leptin might cause melanoma growth through increased NO production and circulating endothelial progenitor cell (EPC) numbers and consequently induced tumor angiogenesis [96].

Additional data showed that leptin signaling deficient mice (*ob/ob* and *db/db*) lacking functional leptin or OB-R, respectively, have reduced incidence of spontaneous and oncogene-induced mammary tumors. These mutant mice are obese and insulin-resistant, but their progeny produced by crossing with mammary tumor-prone mice (MMTV-TGF-α) showed no mammary tumors despite of their body weights were significantly heavier than those from lean groups. In sharp contrast, the incidence of mammary tumors in MMTV-TGF-α/*ob^−^/ob*^+^ (67%), *ob^+^/ob*^+^ (50%) and, *db^−^/db*^+ ^ (69) and *db^+^/db^+^* (82%) was significantly higher. An interesting observation showed the absence of duct formation and branching for MMTV-TGF-α/ *db^−^/db^−^* mice [97,98]. This data demonstrated the importance of leptin biological effects on mammary tissue formation. Indeed, leptin is necessary for normal mammary gland development. However, leptin and OB-R are expressed in low levels on epithelial cells of normal human mammary glands [12].

Interesting data supports the hypothesis that increased adipose tissue mass and adipokines, mainly VEGF, in postmenopausal obesity promoted tumor angiogenesis and breast cancer progression. This data assessed the epidemiological evidence that correlated postmenopausal obesity and breast cancer and confirmed that VEGF was a major angiogenic factor in breast cancer. However, the specific role of other angiogenic factors such as leptin, was not addressed in this study [99]. Therefore, different tumor types could be more or less susceptible to obesity-induced signals, including leptin. Additional research is needed to establish the importance of leptin, insulin and related factors to the paracrine control of tumor angiogenesis.

Leptin signaling induced VEGF and VEGFR2 expression before hypoxic conditions [89]. This data suggested that increased levels of leptin in the tumor microenvironment provided a distinctive advantage to tumor cells, expressing high levels of OB-R, over normal cells, expressing low amount of OB-R, to growth. Remarkably, the inhibition of leptin signaling via leptin peptide receptor antagonist 2 (LPrA2) significantly reduced VEGF/VEGFR2 expression and angiogenesis in human xenografts, estrogen responsive, MCF-7 and non-responsive, MDA-MB231-triple negative breast cancer cells, and mouse mammary tumors. LPrA2 effects paralleled a significant reduction of tumor growth [100,101,102]. Furthermore, PEG-LPrA2 inhibition of leptin signaling in diet-induced-obesity (DIO) mice treated with a carcinogen, 7,12-dimethylbenz[α]anthracene, (DMBA) delayed the onset of mammary tumors and reduced tumor growth. LPrA effects were found together with a significant decrease of leptin-angiogenic targets: VEGF/VEGFR-2, hypoxia HIF-1α, NF-κB p105, IL-1R tI and Notch [100,103]. The use of a different leptin receptor antagonist peptide in a mouse model using MDA-MB231 cells also resulted in a significant reduction of tumor growth [104].

**Figure 1:**
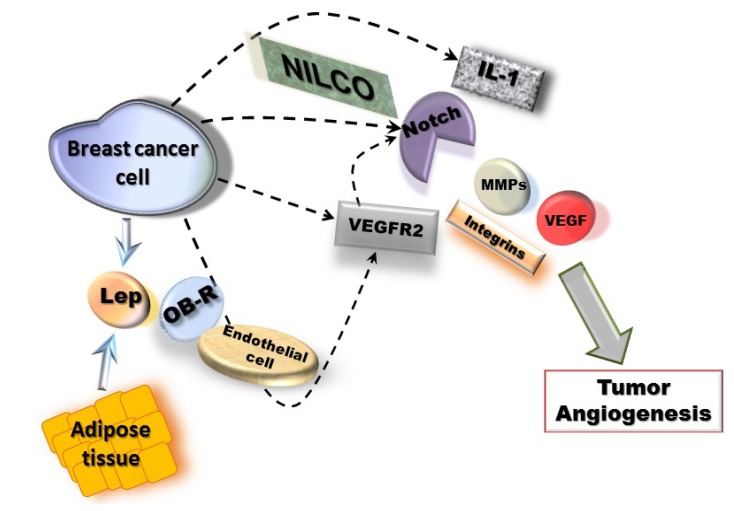
Mechanisms for leptin-induced effects in breast cancer angiogenesis*.* Leptin signaling could be linked to NILCO (Notch, IL-1, leptin crosstalk outcome) [116] and upregulation of pro-angiogenic factors (VEGF/VEGFR2, MMPs and integrins) [34,89]. These leptin actions could involve two waves, a short-term wave, where leptin/OB-R activation could directly transactivate VEGFR2 [45] in endothelial cells and, a long-term wave involving NILCO, MMPs and adhesion molecules in breast cancer cells [116].

Molecular mechanisms of leptin pro-angiogenic actions in breast cancer could involve two waves, a short-term wave that directly transactivates VEGFR2 in endothelial cells and, a long-term wave inducing the upregulation of MMPs/TIMPs, integrins and NILCO (Notch, IL-1 and leptin crosstalk outcome) in breast cancer cells, which positively regulates VEGF/VEGFR2 expression (Figure 1). NILCO could represent the integration of developmental, pro-inflammatory and pro-angiogenic signals critical for leptin-induced breast cancer cell proliferation/migration, tumor angiogenesis and breast cancer stem cells.

### 5.1. Leptin Regulation of VEGF and VEGFR2

Leptin is an upstream regulator of VEGF and VEGFR2 in cancer, including breast cancer [37]. The biological significance of leptin-induced upregulation of VEGF and VEGFR2 expression (at protein and mRNA levels) was investigated in breast cancer cells responsive (4T1 and MCF-7; ER^+^) and unresponsive to estrogen (MDA-MB231; ER^−^) and derived tumor-xenografts hosting by mice treated with a specific leptin antagonist (pegylated, PEG-LPrA2). Leptin significantly increased the levels of VEGF and upregulates the transcriptional expression of VEGF and VEGFR2 that was abrogated by PEG-LPrA2 treatment. Mice treated with PEG-LPrA2 had diminished expression of VEGF/VEGFR2, OB-R, leptin, IL-1R tI, PCNA and cyclin D_1_ and showed reduced growth of 4T1, MCF7 and MDA-MB231 tumors [101,102].

Leptin signals also impacted tumor stroma, noncancerous cells including fibroblasts, immune and endothelial cells, ability to produce VEGF. PEG-LPrA2 treatment decreased levels of human and mouse VEGF and leptin in MCF-7 ER^+^ breast cancer xenografts. This could explain in part the increased effectiveness of PEG-LPrA2 treatment in reducing tumor growth in MCF-7 ER^+^ when compared to MDA-MB231 ER-breast cancer xenografts [102]. Importantly, PEG-LPrA2 treatment did not affect levels of mouse leptin in plasma, suggesting that this compound did not interfere with systemic leptin metabolism. Indeed, no significant differences on body or carcass weights were found between normal-weighted mice hosting breast cancer xenografts and those treated with PEG-LPrA2 [102].

Leptin also induced VEGF/VEGFR2 in other types of cancer cells. Leptin induced VEGF in mouse colon cells carrying the Apc Min mutation, mutated genotype of adenomatous polyposis coli tumor suppressor gene for colon cancer. Conditioned media from mouse colon cells treated with leptin induced HUVEC cell proliferation, chemotaxis, upregulation of adhesion proteins and cell-signaling activation via NFκB nuclear translocation and DNA binding [105].

Comprehensive mechanisms for leptin upregulation of VEGF/VEGFR2 transcriptional expression in breast cancer cells have been reported [89]. VEGF promoter deletion and siRNA analyses showed that leptin signaling regulates VEGF in breast cancer mainly through HIF-1α and NFκB in normoxic conditions. Leptin activation of HIF-1α was mainly linked to canonic, MAPK, PI-3K, and non-canonic, PKC, JNK and p38 MAP, signaling pathways. Similarly, leptin activation of NFκB involved non-canonic signaling pathways, JNK, p38 MAP and to lesser extent PKC. Moreover, leptin-induced SP1 induces VEGF, but AP1 was not involved and AP2 repressed leptin-induced increase of VEGF [89]. Furthermore, leptin-induced regulation of VEGF/VEGFR2 in breast cancer also involved the activation of Src and Gbr2/Gab2/STAT3 and their crosstalk to Rho-GTPases. Indeed, leptin activation of Rac1 induces VEGF/VEGFR2 and promoted a negative feedback, which in turn downregulated activation of Src/Gbr2/Gab2/STAT3. These data suggested a high complexity of signaling crosstalk is involved in leptin regulation of pro-angiogenic factors and breast cancer growth [106].

Leptin was recently reported to induce the transactivation of VEGFR2 in endothelial cells deprived of VEGF. Leptin-induced transactivation of VEGFR2 significantly increased endothelial cell proliferation and angiogenic differentiation *in vitro* EC [45]. Leptin induced rapid phosphorylation of VEGFR2 (Tyr117) via cyclo-oxygenase 2 (COX-2), p38 MAP and AKT signaling pathways. These leptin effects were independent of VEGF and linked to the induction of the pro-angiogenic phenotype of HUVEC, which were blocked by PEG-LPrA2. Moreover, inhibition of VEGFR2 signaling reduced leptin-induced effects in HUVEC cells. Similarly, the blockade of VEGFR2 or COX-2 activities abolished leptin-driven neo-angiogenesis in a chick chorioallantoic membrane assay [45].

Leptin induced the secretion of VEGF, bFGF, TGF-β in prostate cancer cells [107]. In addition, leptin increased tissue-specific induction of Ang-2 in mouse adipose tissue that was related to the initiation of apoptosis in adipose endothelial cells and adipose tissue regression [108]. As it will further be discussed, leptin-mediated upregulation of IL-1 and Notch systems in breast cancer cells was closely linked to regulation of VEGF and VEGFR2 [11,109].

### 5.2. Leptin Regulation of IL-1

Leptin and IL-1-induced signals can crosstalk in many cells and pathologic conditions. Both cytokines play roles in tumor inflammation, proliferation and angiogenesis. The expression of IL-1 in breast cancer was associated with aggressive tumor phenotype. Moreover, the expression of IL-1 in poorly differentiated ER^−^ breast tumors contributed to the malignant phenotype [110]. Accumulated data suggested that leptin and IL-1 system regulation was bidirectional. Leptin induced IL-1 and vice versa [68,109,111].

IL-1 influenced tumor growth, and metastasis in experimental models and in several tumor types including non-small-cell lung carcinoma, colorectal adenocarcinoma, and melanoma tumor [112]. IL-1 played a key role in the onset and development of the host reaction to invasion, and is an important factor in the initiation of the inflammatory response and immune functions. IL-1 system was overexpressed in cancer and tumor inflammatory cells, which affects carcinogenesis, tumor growth, invasiveness, tumor-host interactions and angiogenesis [113].

IL-1 family comprises two ligands (IL-1α and β), two receptors (type I and II: IL-1R tI and II) and a receptor antagonist (IL-1Ra). It was earlier reported that leptin induced phosphorylation of ERK1/2 and JAK2 was involved in the downregulation of IL-1Ra and release of IL-1 in human pancreatic islets. These leptin effects led to impaired β-cell function, caspase-3 activation, and apoptosis, which suggested IL-1Ra could provide protection against high levels of leptin and glucose-induced islet IL-1 [114]. In contrast, later observations from human monocytic cells showed that leptin mediates the upregulation of IL-1Ra. However, these leptin effects were not seen in THP-1 monocytes derived from an acute monocytic leukemia patient [115].

Leptin-induced effects are cell-specific. Direct relationships between leptin and IL-1 system have been detected *in vitro* in breast [102,109] and endometrial cancer cells [103]. In breast cancer cells leptin induced the transcriptional and translational expression of IL-1 family of proteins. Leptin-mediated effects were linked to several canonic and non-canonic signals, *i.e.*, JAK2/STAT3, MAPK/ERK 1/2, PI-3K/AKT1, PKC, p38 and JNK [109]. In line with previous reports from human monocytes [115], leptin induced IL-1β and IL-1Ra expression in breast cancer cells, which was related to the phosphorylation of mTOR/4E-BP1. Moreover, leptin-induced upregulation of IL-1α promoter was linked to SP1 and NF-κB transcription factors. Remarkably, leptin’s effects were partially mediated by IL-1/IL-1R Type I (tI) signaling [109]. Conversely, leptin only induced IL-1R tI in human endometrial cancer cells, An3Ca, SK-UT2 and Ishikawa cells, compared to non-malignant cells, HES and primary human endometrial cells, through a mechanism involving JAK2, PI-3K and MAPK/mTOR signaling pathways. Interestingly, IL-1β was only increased by leptin in benign primary epithelial endometrial cells [103]. These actions of leptin were closely related to the upregulation of VEGF/VEGFR2, which was partially abrogated by inhibition of IL-1 signaling. Overall, leptin’s effects on pro-angiogenic molecules were more evident in malignant *versus* benign cells. This may imply that there is an underlying shift in leptin-induced cell signaling pathways in cancer cells.

*In vivo* data also show that leptin induced IL-1R tI, VEGF and VEGFR2 as well as growth of breast tumors induced by a carcinogen, 7,12-dimethylbenz[a]anthracene (DMBA) in DIO mice [100] and was reported earlier in human breast cancer xenografts [102]. These mice exhibited low tumor growth and reduced expression of IL-1R tI and VEGF/VEGFR2 via treatment with PEG-LPrA2 [100,102]. Additionally, data from immunohistochemical analysis of breast cancer tissue array (n = 67 invasive carcinomas) suggested co-expression of IL-1R tI, leptin/OB-R and VEGF in cancer and stroma cells [62]. These data suggest that leptin and IL-1 system crosstalk is involved in breast cancer angiogenesis and growth.

### 5.3. NILCO: Notch, IL-1, Leptin Crosstalk Outcome

The expression and activation of Notch is a hallmark of breast cancer, which correlates to increased tumor angiogenesis, poor prognosis and survival. Notch signaling has an extensive crosstalk to diverse oncogenic signals in breast cancer. Leptin was recently added to the list of Notch regulators in breast cancer [116].

Notch is a conserved embryonic signal involved in vasculogenesis and angiogenesis, which influences various cell programs including proliferation, differentiation, and apoptosis. Ligands and receptors of Notch are membrane-bound proteins. Cell adjacent ligands bound to Notch expressed on neighboring cells triggered successive proteolytic cleavages of Notch cytoplasmatic region via TACE (ADAM)/γ-secretase. This proteolytic process released Notch intracellular domain (NICD), which translocated into the nucleus and removed corepressors from RBP-Jk(CSL/CBF1/Su (H)/Lag1, a transcription factor). This allowed RBP-Jk to recruit a coactivator complex composed of Mastermind (MAM) and several transcription factors to transcriptionally activate Notch target genes involved in the inhibition of apoptosis, activation of the cell cycle, survival and stimulation of angiogenesis. In the absence of activated Notch, the DNA binding protein RBP-Jk formed a complex with corepressor molecules that inhibited the transcription of Notch-target genes [87].

Notch pathway is cell/context dependent and influences cell fate. However, scarce information on leptin-Notch interactions is available. Leptin was found to regulate the expression of Notch4 receptor and its ligand, JAG1 in human endothelial and blood CD34^+^ cells [117]. More recently leptin was shown to activate Notch signaling pathway in breast cancer and endothelial cells under normoxic conditions [116,118]. Remarkably, in breast cancer leptin upregulated Notch1-4/JAG1/Dll-4, Notch target genes: Hey2 and survivin, together with IL-1 and VEGF/VEGFR2. These leptin effects were linked to the activation of several signaling, JAK2/STAT3, MAPK, PI-3K/mTOR, p38 and JNK, and specific transcription factors, NFκB, Sp1 and HIF-1α [116]. Leptin-induced Notch was linked to the increase of breast cancer cell proliferation, migration, and angiogenic differentiation of endothelial cells [116,118].

Interestingly, leptin effects on cell proliferation/migration and pro-angiogenic factors, Notch, IL-1 and VEGF/VEGFR2, were abrogated by a γ-secretase inhibitor, an essential protease activating membrane-bound receptors, DAPT, siRNA against CSL and by the blockade of IL-1R tI. This data assessed a previous report on inhibition of leptin-induced VEGF/VEGFR2 via IL-1 signaling blockade [109].

Overall, leptin induction of proliferation/migration and upregulation of VEGF/VEGFR2 in breast cancer cells were related to an intact leptin-Notch-IL-1 signaling axis. Therefore, novel crosstalk between the molecules, Notch, IL-1 and leptin crosstalk outcome, (NILCO), was suggested to play an essential role in the proliferation/migration and expression of pro-angiogenic molecules in breast cancer [116]. NILCO could represent the integration of developmental, pro-inflammatory and pro-angiogenic signals critical for leptin-induced cell proliferation/migration.

Additionally, Notch activation could enhance the oncogenic potential of other molecules with an important role in obesity and breast cancer angiogenesis, *i.e.*, insulin [9,87] and IGF-1. Notch activated IGF-1 by stimulating IGF-1R pathway and cancer cell survival [119]. Thus, the relationship between adipocyte size, insulin sensitivity and angiogenesis remained uncertain [120]. Moreover, IGF-1 or insulin regulation of the Notch pathway has not been reported.

### 5.4. Leptin Regulation of MMPs and Integrins

Angiogenesis mechanisms involve the specific proteolytic cleavage of the basement membrane and extracellular matrix via MMPs and other proteases and, the degradation and synthesis of several adhesion molecules [18]. Leptin stimulation of circulating angiogenic cells involved Src kinase phosphorylation of αvβ5 integrins, which was linked to JAK2 and phospholipase C (PLC) γ activation. These leptin actions induced the formation of new vessels in chicken embryo chorioallantoic membrane and improved neovascularization of ischemic murine hind limbs [35]. Similarly, leptin activation of JAK2/STAT3 impacted the expression of αvβ5 and α4 integrins in human endothelial progenitor cells (EPCs), which affected vascular remodeling. Therefore, leptin enhanced EPC capacity to promote vascular regeneration *in vivo* [121]. In human prostate cancer and chondrosarcoma cells, leptin increased expression of αvβ3 via IRS-1/PI3K/Akt, and NFκB signaling cascades promoting cell migration [122,123]. Moreover, in a well-characterized human endometrial adenocarcinoma cell line, Ishikawa cells, leptin increased avβ3 expression [21,23,34].

Leptin can further contribute to tumor angiogenesis via induction of MMPs and integrin expression. In invasive human cytotrophoblast, whose invasive potential closely resembles cancer cells, leptin induced α5 and α6 integrins and MMP9 activity [34]. In human atherosclerotic lesions, rat cornea, HUVECs and human coronary artery smooth muscle cells (HCASMCs) leptin mediated angiogenesis by increasing MMP2 and 9 and TIMP1 and 2 [36]. Furthermore, glioma cells expressed high levels of leptin and leptin receptors than nonmalignant astrocytes and increase proliferation, migration and secretion of several MMPs, MMP2, 9 and 13 by leptin activation of p38 MAP kinase and NFκB pathways [124]. Leptin also induced migration of gastric cancer cells via enhanced interaction of MT1-MMP with kinesin 1B, a microtubule plus end-directed monomeric motor protein, (KIF1B) [125]. Conversely, in human hepatic stellate cells (LX-2) leptin repressed the basal level of constitutive MMP1 mRNA and its promoter activity that was related to JAK/STAT, ERK1/2 and p38 signaling pathways. However, in these studies leptin’s impact on tumor angiogenesis was not investigated.

Taken all together, data strongly suggest that leptin signaling plays an important role in cancer development and/or progression that could be mechanistically linked to the upregulation of pro-angiogenic and pro-proliferative factors (see Figure 1).

## 6. Pathway Studio 9 Analysis of Leptin Pro-Angiogenic Networks in Breast Cancer

**Figure 2:**
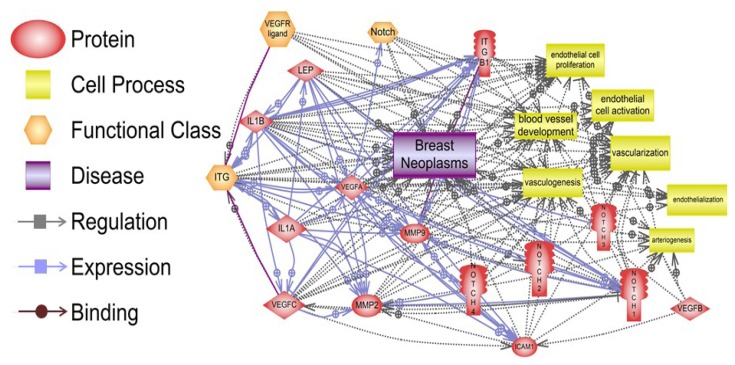
Relationships between pro-angiogenic effects of leptin signaling and breast neoplasms, vascularization, and endothelial cell function. Many signaling pathways show the connections between regulation, expression and binding of leptin, IL-1, adhesion molecules (ICAM and integrins), Notch, VEGF/VEGFR and MMPs and, their impact on vessel development and endothelial cell function, which are essential for breast neoplasm development. The summary of the data processed by the Program Studio 9 [19] and specific molecular and cellular relationships can be found in the supplementary material. Notes: ITG (integrins); ITGB1 (integrin β1); IL-1A and B (IL-1α and β); Lep (leptin); ICAM (CD54, intercellular adhesion molecule 1).

Pathway Studio 9 software (Ariadne Genomics, Rockville, MD, USA) was used to analyze *in silico* the potential targets of leptin pro-angiogenic signals and relationships to oncogenic factors [31], which could also impact breast cancer angiogenesis. Figure 2 depicts the various relationships between these molecules in breast cancer. One hundred sixty three relationships that include regulation, expression and binding of main leptin targets were detected in breast neoplasms. The proteins: leptin, IL-1, integrins, Notch and VEGF/VEGFR and vascularization and/or endothelial cell activation showed many relationships in breast neoplasms. These results are included in the supplemental material (S1). Among several leptin relationships with cell processes involving cancer and angiogenesis, changes of leptin/OB-R levels were related to breast cancer (38 references). Leptin/OB-R signaling was also related to regulation of vascularization and vasculogenesis (118 references), endothelial cell function, proliferation and vessel development (39 references). Numerous relationships between leptin/OB-R and angiogenic molecules (IL-1, Notch and VEGF/VEGFR) were detected (see Supplementary Material). The results of this analysis underline the many facets of leptin pro-angiogenic effects in cancer.

## 7. Conclusions and Perspectives

Pleiotropic effects of leptin in breast and other cancers involved the increase of cell proliferation linked to leptin-induced expression of cell cycle proteins and regulators and, anti-apoptotic and inflammatory factors. However, leptin pro-angiogenic actions are relevant for breast cancer progression. NILCO appears to be a central process involved in leptin’s effects on tumor angiogenesis, which is mainly driven through direct activation of VEGFR2 and upregulation of VEGF/VEGFR2 expression. Failure of initially promising anti-angiogenic therapies [126] and the resistance to anti-angiogenic drugs mainly targeting VEGF/VEGFR2, are currently relevant problems jeopardizing the successful outcomes of these breast cancer treatments [127]. Compensatory and redundant effects of other angiogenic factors, *i.e.*, leptin and its crosstalk partners IL-1 and Notch, could be paramount for these failures. The rising incidence of obesity and fatality with breast and other cancers make it essential to identify patient groups that would benefit for more specific anti-angiogenic therapies. Forthcoming identification of potential biomarkers for anti-VEGF/VEGFR2 drug effectiveness could include leptin pro-angiogenic signaling and molecular targets. Therefore, combining anti-VEGF/VEGFR2 therapies with leptin signaling antagonists could be a potentially novel way to treat breast cancer.

## Supplementary Files

Supplementary File 1 Supplementary (XLSX, 53 KB)
